# The Prevention of Disease

**Published:** 1899-04

**Authors:** S. A. Buchanan

**Affiliations:** Philadelphia


### SELECTIONS.

### The Prevention of Disease.

BY S. A. BUCHANAN, M.D., OF PHILADELPHIA.

It is quite a true saying that "An ounce of prevention is worth a pound of cure." The German nation has long been cognizant of the fact that there is more truth than fiction in the above statement. Some of the primary schools in Germany have their own physician. He watches over the class-rooms, and is there to show that questions of warming, ventilation, lighting and cleaning have entered into the kingdom of science. Once a fortnight he gives instructions to every class in the school on sanitation, and is practically the health officer of the whole establishment.

It is, no doubt, humilating to us to acknowledge that the German nation has taken a step in advance of our rapid and progressive nation in sanitary science as well as medicine.

The Philadelphia Board of Education, when diphtheria was so prevalent last winter, considered the feasibility of appointing a physician to act in a similar capacity as the German physician does in his school, but as the epidemic subsided they became indifferent. Not long since they were confronted with the question of how to best disinfect our school rooms daily at the least expense. Some suggested that the janitor be instructed how to do it; others contended that his knowledge of such matters would be too limited. It is gratifying to see our Board of Education agitating such important questions which are of so much vital moment, and shows to the public they are abreast of the times.

The answer to the above problem would be either to appoint a physician, whose duty would be to make daily inspection of each scholar on admission, disinfect the school room, either before opening or after dismissal of school; to attend to all vaccination of children who are too poor to pay for having it done by the family physician, and thus do away with our vaccine physician; to give lectures upon sanitary science or the laws of health, or instruct our health inspectors to act in a similar position with an increased salary. Surely "in time of peace is the time to prepare for war," and so it is with disease.

It is interesting to note how sanitary science is associated with the life history of every nation, and how largely it entered into the history of civilization.

The Germans have evidently struck the right key-note in enlisting the services of the physician in the continuous efforts to inculcate the best rules of life and action, and instilling in the minds of the youth the importance of the knowledge of sanitary science. But while it is the special province of the medical profession, as guardians of the public health, to study the causes of physical deterioration and disease, and to point out how far these causes may be controlled or averted, the general well-being of the people must mostly depend on their education of sanitary science and laws of health during their youth. More has been accomplished by dissiminating a knowledge of these fundamental principles of hygiene among our youth than by police regulations or legislative enactments. The teaching of physiology and the laws of health have already been productive of much good. Last winter, when diphtheria was so prevalent, considerable anxiety and loss of life would have been avoided if precaution had been taken in our schools.

As I have said, sanitary science enters into the history of every nation. We have exemplified in the Mosaic code of the Jewish race that their comparative longevity was due to the observance of its instructions.

Or let us glance at those grand old Greeks, who have left to posterity such a rich store of philosophy, literature and art. It is true that though their sanitary code, propounded by Lycurgus, was severe and cruel, yet the means which they adopted in the care and cultivation of their bodily physique and the development of their intellectual faculties made them the civilizers of the old world and exemplars of modern times. Then the Romans, the other powerful nation of antiquity, we find that, though they contributed but little to sanitary science, they have left some wonderful examples of sanitary engineering on a large scale. Their Cloaco Maxima, for example, and the aqueduct for conveying water from the hillside, some thirty miles from Rome, were works of such stupendous magnitude that have seldom been equalled or surpassed at the present day.

If our city fathers were as familiar with sanitary science as the Romans we would no longer be obliged to quench our thirst with the filthy water of the Schuylkill River; our system of underdrainage would be more complete; our alleys and gutters would be free from filth and garbage, which are such favorable fields for the development of disease germs. I would suggest that our city fathers go to the Romans and not to the ants to learn lessons of wisdom.

I do not speak of these matters from a pessimist's standpoint, for I feel proud of the city of my birth as well as my country.

I am told that in Dresden there are public lavatories every three or four squares. I contend that if we possessed more of such public conveniences and purer water many a man would not be so readily induced to drink intoxicants. Our Women's Christian Temperance Union have done noble work in erecting public fountains, thus alleviating the thirst for man and beast, but there is a broader field for them yet, and they can still have their names indelibly inscribed upon the tablet of fame by having erected such monuments, which are of absolute necessity as well as a convenience to mankind. When a man is seized with the desire to defecation or urination there is no other alternative than to go to the saloon, where they possess a closet, and respond to nature's call. In return for this convenience a man feels under obligation, whether he really desires it or not, to purchase a glass of beer, and if, by chance, he should see any of his friends there he will invariably treat the crowd, and they through courtesy respond to his generosity until several are indulged in, and as a result the man is intoxicated before he is aware of it. This is an inexhaustible subject, but in my daily practice I see so many evils resulting from the neglect of sanitation that one who is interested in the health of our city cannot fail to call the attention of the public mind to it.

I am rarely ever called to treat a case of diphtheria unless I trace its origin to overfilled cesspools in the yard, or a choked water closet, or a filthy cellar, dug in filthy soil. Of course, there are a few exceptions.

Let us always impress upon the minds of the masses, as well as the foreign element, who are overcrowded in tenement houses, that "Cleanliness is next to Godliness," and we will be free from pestilence.—*Ex.*

